# The Age-Related Risk of Co-Existing Meningitis in Children with Urinary Tract Infection

**DOI:** 10.1371/journal.pone.0026576

**Published:** 2011-11-09

**Authors:** Marc Tebruegge, Anastasia Pantazidou, Vanessa Clifford, Gena Gonis, Nicole Ritz, Tom Connell, Nigel Curtis

**Affiliations:** 1 Department of Paediatrics, The University of Melbourne, Parkville, Victoria, Australia; 2 Infectious Diseases Unit, Department of General Medicine, Royal Children's Hospital Melbourne, Parkville, Victoria, Australia; 3 Murdoch Children's Research Institute, Parkville, Victoria, Australia; 4 Department of Microbiology, Royal Children's Hospital Melbourne, Parkville, Victoria, Australia; Columbia University, United States of America

## Abstract

**Objective:**

The primary aim of this study was to determine age-stratified rates of co-existing bacterial meningitis in children with urinary tract infection (UTI). The secondary aims of this study were to determine the causative pathogens of UTI, and the clinical features and outcome of children with co-existing meningitis.

**Methods:**

Analysis of data collected over a nine-year period at a tertiary pediatric hospital in Australia. Study population: children below 16 years of age with culture-confirmed UTI and a paired CSF sample.

**Results:**

A total of 748 episodes in 735 cases were included in the final analysis. The commonest pathogens causing UTI were *Escherichia coli* (67.4%), *Enterococcus faecalis* (8.4%), *Klebsiella oxytoca* (3.5%) and *Klebsiella pneumoniae* (3.5%). Only two (1.2%; 95% CI: 0.15–4.36%) of 163 neonates (between 0 and 28 days of age) with UTI had co-existing meningitis. Both presented with pyrexia, irritability and lethargy, and recovered uneventfully with antibiotic treatment. There were no cases of co-existing meningitis among 499 infants (between 29 days and 12 months of age) with UTI (95% CI: 0.00–0.74%), or any of the 86 children aged 12 months or over (95% CI: 0.00–4.20%).

**Conclusions:**

These findings indicate that clinicians should have a low threshold to perform a lumbar puncture in neonates with UTI, as the risk of co-existing meningitis is not insignificant in this age group. In contrast, beyond the neonatal period, the risk is small and a more selective approach is warranted.

## Introduction

Urinary tract infection (UTI) is common in infants and young children. Between 1% and 15% of young children presenting to hospital with pyrexia have an underlying UTI [1], [2]. Previous studies show that bacteremia in association with UTI is not uncommon in infants, occurring in 4% to 10% [3]–[7]. As meningitis is generally the result of dissemination of bacteria via the bloodstream and subsequent penetration of the blood-brain barrier [8], [9], it has been suggested that young children with UTI are therefore at higher risk of co-existing meningitis.

A study by Bergstrom *et al.* published in 1972, which is frequently referred to in this context, suggests that co-existing meningitis is very common in infants with UTI [10]. The authors reported that co-existing bacterial meningitis was present in six (19.4%) of the 31 infants with UTI who had a lumbar puncture. Subsequent studies have reported considerably lower estimates for the risk of co-existing meningitis in children presenting with UTI [3]–[7], [11]–[19]. However, the majority of studies have significant limitations, including small sample size [4], [6], [13], [15]–[19] and absence of a precise definition for UTI [12], [17]. Furthermore, in the majority of previous studies, the study population exclusively comprised infants younger than three months of age, [3]–[6], [12], [14]–[18] and therefore only limited data exist regarding older infants, as well as children outside infancy.

Accurately determining the rate of co-existing meningitis in children with UTI is clinically highly relevant. In the absence of reliable data, it remains uncertain whether young children with UTI should always have a lumbar puncture to rule out co-existing meningitis [20]. The age after which the risk of co-existing meningitis becomes negligible is also unknown. Identification of co-existing meningitis is critically important, as failure to diagnose central nervous system (CNS) infection may result in partial or incomplete treatment, particularly in view of the fact that several national guidelines suggest that uncomplicated UTI in children can be treated with short courses of oral antibiotics, some of which have poor CNS penetration [21], [22].

The primary aim of our study was to determine accurate, age-stratified rates of co-existing meningitis in children with culture-proven UTI. Secondarily the study aimed to determine the spectrum of bacteria causing UTI in children, and to describe the clinical features and outcome of children with co-existing meningitis.

## Methods

### Study design and setting

The study was conducted at the Royal Children's Hospital Melbourne, the largest pediatric referral center in the state of Victoria, Australia (estimated population 1.19 million children between 0 and 18 years of age). We analyzed data collected over a nine-year period (1^st^ Jan 2001 to 1^st^ Jan 2010). The following data were extracted from the hospital microbiology department database: patient age, date and type of sample, and bacterial culture results. In patients who potentially fulfilled the case definition of UTI and co-existing meningitis, additional clinical and laboratory data were extracted from the patient's record by one of the investigators.

### Inclusion criteria and definitions

Patients between one day and 16 years of age were eligible for inclusion if they had a positive urine culture and a cerebrospinal fluid (CSF) sample obtained within 48 hours of the urine sample being collected. Cases were identified by matching all children with a positive urine culture result during the study period with CSF culture results.

In this study, ‘neonate’ was defined as a patient between 0 and 28 days of age; ‘infant’ was defined as a patient between 29 days and 12 months of age. UTI was defined as: i) a pure growth of a single bacterial pathogen with ≥10^2^ colony forming units (CFU)/ml from a urine sample obtained by suprapubic aspirate or catheterization, ii) ≥10^3^ CFU/ml from a clean catch urine sample or, iii) ≥10^5^ CFU/ml from a bag urine sample. In cases with more than one UTI, these were included in the analysis as separate episodes if they occurred more than 14 days apart. For each episode, only the result of the first positive urine culture was included in the data analysis. In the event that two different bacterial pathogens were isolated from two separate urine samples during one episode, the culture result of the more reliable sample was included in the final analysis (suprapubic aspirate>catheter urine>clean catch urine>bag urine).

CSF pleocytosis was defined as a CSF white blood cell (WBC) count on microscopy of >19/µl during the first month of life, and >9/µl thereafter, based on recently published data [23]. In blood-stained CSF samples, the WBC count was corrected according to the number of red blood cells using a fixed ratio of 1∶500 [24]. Definite meningitis was defined as the growth of a single bacterial pathogen from a CSF sample. Co-existing meningitis was defined as a positive CSF culture with a pure growth of the same bacterial pathogen that was isolated from the patient's urine culture. *Micrococcus* species, *Bacillus* species, coagulase-negative staphylococci and viridans streptococci isolated from CSF were considered to be contaminants [25], unless the same organism was simultaneously isolated from urine.

### Exclusion criteria

Episodes with urine cultures that showed mixed growth (ie two or more organisms in one sample) were excluded, as these are likely to represent contaminated samples. Episodes with urine samples growing fungal organisms (ie possible fungal UTI) were also excluded.

### Assays for antimicrobial activity

CSF samples were routinely tested for antimicrobial activity to identify samples which may yield false-negative culture results due to the presence of antibiotics administered prior to the CSF being obtained, as previously described with minor modifications [26]. Briefly, the antimicrobial activity assays were done by adding one drop of CSF to Mueller-Hinton agar plates (Oxoid, Adelaide, Australia) enriched with a fully sensitive strain (ATCC 6633) of *Bacillus subtilis* (Chrisope Technologies, Louisiana, USA). Presence of antibiotics leads to inhibition of bacterial growth resulting in a zone of clearing around the inoculated CSF, which is assessed after overnight incubation at 35°C.

### Data analysis

Data analysis and the construction of the figures were done using Prism (GraphPad Software Inc., La Jolla, California) and Excel (Microsoft, Redmond, Washington). Nonparametric statistics were used for age-related data, as these data were not normally distributed. The ‘exact’ method was used to calculate 95% confidence intervals (CI). A two-sided chi-square test was used to compare the rate of co-existing meningitis between different age groups.

### Ethical approval

The study was approved by the Human Research Ethics Committee of the Royal Children's Hospital Melbourne (HREC 30062A).

## Results

A total of 735 cases who fulfilled the inclusion criteria were identified. Thirteen of these cases had two episodes of UTI during which CSF had been obtained. None of the cases had three or more episodes. Therefore, a total of 748 episodes were included in the final analysis. Table 1 shows the demographic characteristics of the study population. The majority of cases (88.5%) were below the age of 12 months.

**Table 1:** Baseline characteristics and rate of definite co-existing meningitis according to age group.

**Age (months)**	
median	2.2
IQR	1.1–4.6
range	0.1–191.2
**Gender**	
male∶female	1.8∶1

Age group	Number of episodes(n = 748)	Number of cases with co-existing meningitis(n = 2)
<1 month	163 (21.8%)	2 (1.2%; 95% CI: 0.15–4.36%)
1–2 months	304 (40.6%)	0 (95% CI: 0.00–1.25%)
3–5 months	129 (17.2%)	0 (95% CI: 0.00–2.82%)
6–11 months	66 (8.8%)	0 (95% CI: 0.00–5.44%)
12–23 months	32 (4.3%)	0 (95% CI: 0.00–10.89%)
24–59 months	21 (2.8%)	0 (95% CI: 0.00–16.11%)
≥5 years	33 (4.4%)	0 (95% CI: 0.00–10.58%)

CI: confidence interval; IQR: interquartile range.

In 607 (81.1%) episodes, urine and CSF samples had been obtained on the same day, in 114 (15.2%) episodes within one day, and in 27 (3.6%) episodes within two days. The most common type of urine sample was catheter urine (31.1%), followed by clean catch urine (28.2%), and suprapubic aspirates (20.9%) (Table 2).

**Table 2:** Urine sampling technique and urine culture isolates.

Type of urine sample(n = 748)	
Suprapubic aspirate	156 (20.9%)
Catheter	233 (31.1%)
Clean catch	211 (28.2%)
Urine bag	7 (0.9%)
Nephrostomy/ureterostomy	6 (0.8%)
Not stated	135 (18.0%)

Pathogen isolated in urine culture(n = 748)	
*Escherichia coli*	504 (67.4%)
*Enterobacter faecalis*	63 (8.4%)
*Klebsiella oxytoca*	26 (3.5%)
*Klebsiella pneumoniae*	26 (3.5%)
*Enterobacter cloacae*	19 (2.5%)
*Proteus mirabilis*	18 (2.4%)
Group B streptococci	17 (2.3%)
*Pseudomonas aeruginosa*	13 (1.7%)
*Staphylococcus aureus*	12 (1.6%)
*Citrobacter* species	9 (1.2%)
*Serratia marcescens*	5 (0.7%)
Other	36 (4.8%)

The spectrum and frequency of bacterial pathogens isolated from urine cultures are summarized in Table 2. *Escherichia coli* was by far the commonest isolate, accounting for 67.4% of positive urine cultures, followed by *Enterococcus faecalis* (8.4%), *Klebsiella oxytoca* (3.5%) and *Klebsiella pneumoniae* (3.5%).

### Cases with UTI and definite co-existing meningitis

There were two cases of UTI with definite co-existing bacterial meningitis. Case 1 was a male neonate who had been diagnosed with bilateral renal dysplasia antenatally. On day 15 of life he presented with pyrexia, poor feeding, irritability and lethargy. The serum C-reactive protein (CRP) concentration was elevated (36 mg/L); the peripheral blood WBC was within normal limits. A catheter urine sample was obtained and a lumbar puncture was done at the same time. A pure growth of *Staphylococcus aureus* was detected in both the urine and the CSF sample. The patient received two weeks of intravenous flucloxacillin and gentamicin. Case 2 was a 19-day-old male neonate who presented with pyrexia, poor feeding, irritability and lethargy. A catheter urine sample was obtained and a lumbar puncture done 12 hours later. The CRP was within normal range (<8 mg/L), but the full blood count revealed neutrophilia (total WBC 12.0×10^9^/L, neutrophils 9.0×10^9^/L). A pure growth of *E. coli* was detected in both the urine and the CSF sample; an assay for *E. coli* K1 antigen in CSF was also positive. The patient received two weeks of ceftriaxone and gentamicin, followed by a further two weeks of ceftriaxone monotherapy. Both patients had an uncomplicated clinical course, and were discharged without evidence of neurological sequelae.

Table 1 shows the rate of definite co-existing meningitis according to age group. The overall rate of definite co-existing meningitis in infants under the age of 12 months was 0.30% (95% CI: 0.04–1.09%); for all ages combined the incidence was 0.27% (95% CI: 0.03–0.96%). There were no cases of definite co-existing meningitis among the 499 infants with UTI that presented outside the neonatal period (95% CI: 0.00–0.74%). Further analysis confirmed that the rate of definite co-existing meningitis was significantly higher in neonates than in infants outside the neonatal period (p = 0.013).

### Description of additional cases with positive CSF cultures

In an additional four patients, pathogenic bacteria were isolated from the CSF sample, but these did not fulfill the case definition for definite co-existing meningitis.

In two cases, it is likely that the children did not have meningitis. In an 8-day-old, previously healthy term neonate, *Enterococcus faecalis* was isolated from urine obtained by suprapubic aspirate. The CSF sample did not show pleocytosis (CSF analysis: WBC 10/µl (neutrophils 0; lymphocytes 10), RBC 18,750/µl, protein 1.18 g/L, glucose 2.6 mmol/L) and no antimicrobial activity was detected. *Micrococcus* species were grown from CSF within 48 hours. The following day, a scanty growth of *E. faecalis* was detected in CSF enrichment cultures. In a 40-day-old infant with congenital posterior urethral valves, a urine sample obtained by suprapubic aspirate grew *E. coli*; the CSF grew scanty mixed *E. coli* and *E. faecalis* (CSF analysis: WBC 8/µl (neutrophils 6; lymphocytes 2), RBC 6,410/µl, protein 0.42 g/L, glucose 2.5 mmol/L). In both cases CSF findings were not suggestive of meningitis making contamination of the CSF sample likely.

In the other two cases the children had bacterial meningitis, but their urine sample was likely contaminated. In a two-month-old infant with Down's syndrome and complex cardiac malformation, a catheter urine sample grew *E. coli*, while group B streptococci were isolated from blood cultures and the CSF. In a 16-month-old child with a ventriculo-peritoneal shunt, *E. coli* was isolated from a urine sample (sampling method not stated on the request form), while CSF cultures grew *S. aureus*.

### Antimicrobial activity assay results

**Figure 1:**
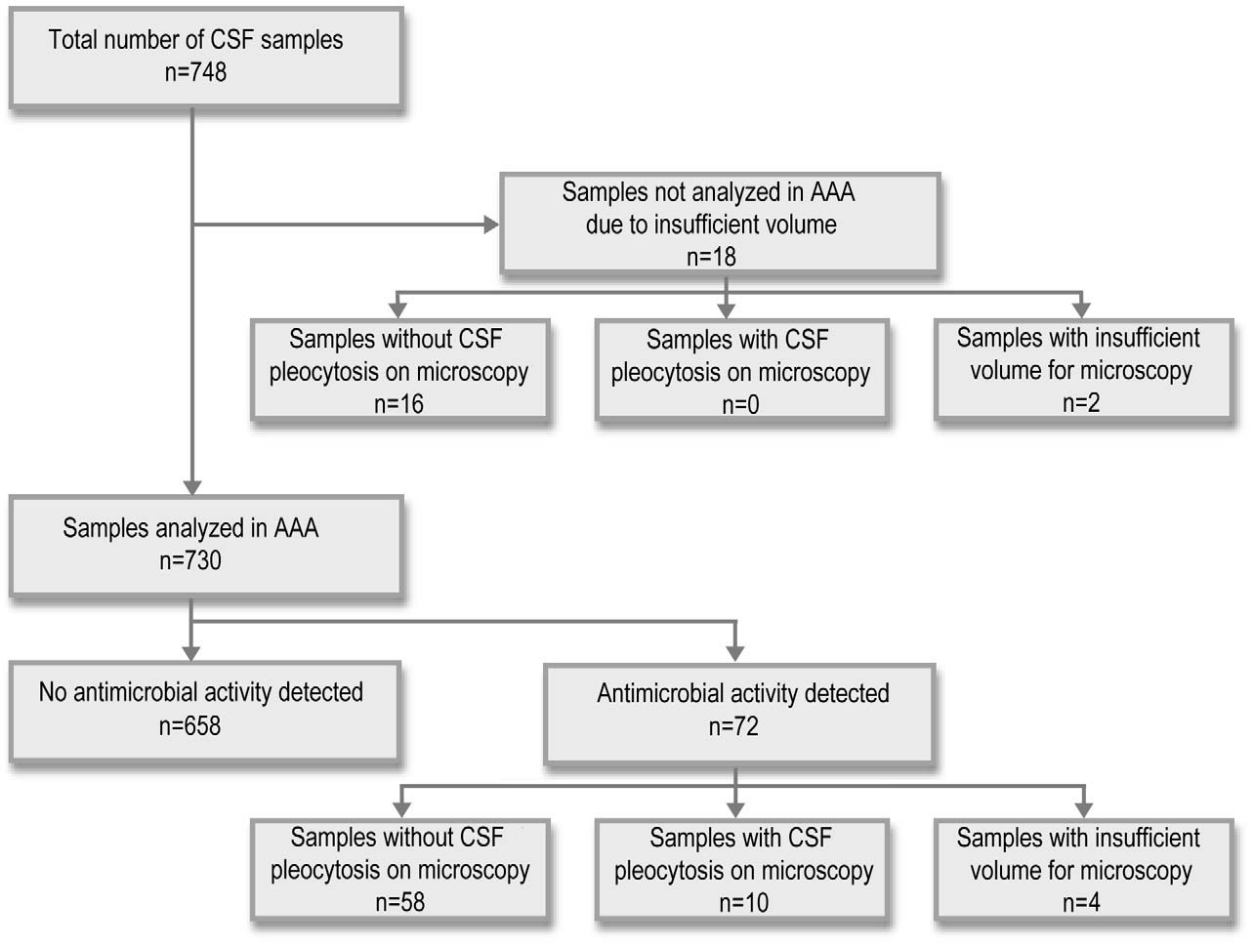
Summary of antimicrobial activity assays done on cerebrospinal fluid samples. Summary of the results of the antimicrobial activity assays (AAA) in relation to cerebrospinal fluid (CSF) microscopy results.

Antimicrobial activity assays were done on the CSF samples from 730 (97.6%) episodes (Figure 1). In 658 (90.1%) of these CSF samples, no antimicrobial activity was detected. In the 72 remaining CSF samples, antimicrobial activity was detected. Four of these samples were clotted and could not be analyzed by microscopy. Of the remaining 68 samples, only 10 had a corrected WBC count consistent with pleocytosis (Table 3). One of these patients was case 2 described above. In five patients, an alternative diagnosis explaining the CSF pleocytosis was identified, comprising meningococcal sepsis/meningitis, ventriculo-peritoneal shunt infection, CNS lymphoma, miliary tuberculosis and cytomegalovirus infection. In none of the remaining four patients were microscopy and biochemical findings characteristic of bacterial meningitis.

**Table 3:** Cerebrospinal fluid findings and diagnoses in patients with CSF antimicrobial activity detected and CSF pleocytosis.

Case	Age(months)	WBC *(/µl)	Neutrophils(/µl)	Lymphocytes(/µl)	RBC(/µl)	Protein(g/L)	Glucose(mmol/L)	Diagnosis
**A**	9.5	7690	7290	400	60	1.49	2.8	Meningococcal sepsis (blood culture: *N. meningitidis*; CSF microscopy: Gram-negative diplococci)
**B**	0.7	1820	1590	230	700	1.62	4.2	Co-existing *E. coli* UTI and meningitis(‘Case 2’ described in results section)
**C**	2.4	1674	1557	117	36	1.00	2.6	VP shunt infection (CSF microscopy: Gram-positive cocci; urine culture: *K. pneumoniae*)
**D**	184.3	620	0	620	30	2.87	<1.2	CNS lymphoma
**E**	2.7	447	183	264	1760	0.14	2.7	
**F**	27.2	144	83	61	24	n.d.	n.d.	Miliary tuberculosis
**G**	63.5	92	15	77	68	1.66	3.2	
**H**	4.1	19	2	17	4250	0.42	3.1	
**I**	10.6	16	0	16	0	1.04	3.0	Cytomegalovirus Infection
**J**	1.1	12	2	10	16	0.88	2.8	

CNS: central nervous system; CSF: cerebrospinal fluid; n.d.: not done; RBC: red blood cell count; VP: ventriculo-peritoneal; WBC *: white blood cell count (uncorrected).

## Discussion

To our knowledge this study is the first to provide age-stratified data on co-existing meningitis in children of all ages with UTI. Our study is considerably larger than any previous study in this area with the exception of the recent study by Schnadover *et al.* that exclusively investigated patients between 29 and 60 days of age [3].

The aforementioned study by Bergstrom *et al.* is frequently quoted as evidence that young children with UTI are at significant risk of co-existing meningitis [10]. However, the limitations of this study have previously been highlighted, and it is likely that it considerably overestimates the risk of co-existing meningitis [6], [14]. Firstly, all urine samples in this study were obtained using urine bags, which are far more prone to contamination than samples obtained by sterile techniques (ie suprapubic aspiration or catheterization) [27]. This may have led to a considerable number of false-positive urine culture results. Consequently, a significant proportion of patients with meningitis could falsely have been classified as having co-existing UTI. Secondly, only 31 (38.8%) of the 80 neonates with UTI in this study had a lumbar puncture. As it is likely that lumbar punctures were predominantly performed on the ‘sicker’-appearing neonates, this could have introduced a significant selection bias and skewed the reported rate of co-existing meningitis even further.

Since the publication by Bergstrom *et al.*, several other studies have investigated co-existing meningitis in children with UTI [3], [7], [11]–[19]. However, the majority are limited by the size of the study population, which in most instances comprised fewer than 200 patients with UTI who had a lumbar puncture [4], [14], [17], [19], or even less than 100 patients [6], [13], [15], [16], [18]. This is a significant limitation, in the light of more recent data suggesting that co-existing meningitis is uncommon in patients with UTI.

The majority of previous studies have exclusively focused on infants under three months of age [3]–[6], [10], [12], [14]–[18], and the available data in older children are limited. In previous reports, the rates of co-existing meningitis in children with UTI varied between 0% [6], [12]–[15], [17], [19] and 2% [18]. However, a further limitation of previous studies is that only few have reported confidence intervals [4], [6], which are critically important for the interpretation of incidence data related to uncommon events. Our data show that the risk of co-existing meningitis in infants with UTI below the age of one month is not insignificant, with the true risk being between 0.15% and 4.36%. In contrast, in infants outside the neonatal age this risk is relatively small (between 0.00% and 0.74%).

Ginsburg and McCracken, who reported a study in 100 infants with UTI, found that the proportion of positive blood cultures correlated inversely with age [13]. Positive blood cultures were observed in 31.4% of cases aged below 1 month, in 18.4% of cases aged 1 to 3 months, and in only 5.6% of cases aged 3 to 12 months. In view of the fact that co-existing bacterial meningitis in patients with UTI is generally the result of bacterial dissemination via the blood stream, this observation further supports the notion that the risk of co-existing meningitis declines significantly with increasing age.

While it could be argued that universal lumbar puncture would be the safest strategy in infants with UTI, this procedure is uncomfortable and can be associated with rare, but potentially serious complications, such as brainstem herniation, introduction of infectious organisms into the CSF and epidermoid tumors [28]–[30]. Based on our data, in conjunction with the results from other studies, we do not believe that universal lumbar puncture in children with UTI outside the neonatal age is necessary. This does not imply that lumbar puncture is not warranted in patients outside the neonatal period who have symptoms or signs indicating possible meningitis.

In our study, both patients with co-existing meningitis were neonates who presented with features suggestive of meningitis, including pyrexia, poor feeding, irritability and lethargy. However, clinical features are insufficiently sensitive to reliably rule out meningitis in neonates and typical meningeal signs are often absent in this age group [9], [31].

It is important to highlight that our study only includes data from patients who had paired urine and CSF results. Patients with UTI who did not have a lumbar puncture were not included, as the presence or absence of meningitis cannot be established. Consequently, our data are more likely to overestimate rather than underestimate the risk of co-existing meningitis, as clinicians are likely to have done a lumbar puncture preferentially on ‘sicker’-appearing children.

The spectrum and frequency of bacterial pathogens causing UTI in our study is consistent with previous pediatric studies, with *E. coli* accounting for more than two thirds of infections [3]–[6], [13], [14], [16], [18], [32], [33]. Similar to other reports *Enterococcus*, *Klebsiella* and *Enterobacter* species were also commonly implicated [3], [5]–[7], [14], [16], [18].

### Strengths and limitations

The strengths of our study include the large sample size, the age-stratification of data and the quality of the urine samples included. More than half of the urine samples were obtained by suprapubic aspirate or catheterization, which are less prone to contamination than other sampling methods [27]. Only a small proportion were documented to be bag urine samples. In addition, we excluded all urine samples with mixed growth to prevent the inclusion of patients with false-positive urine culture results.

Limitations of our study include its retrospective nature. We cannot exclude with absolute certainty the possibility that some patients had received antibiotics prior to the CSF sample being obtained, thereby potentially rendering their CSF culture false-negative, as we did not have data regarding the timing of the lumbar puncture and the initiation of antibiotic treatment. However, in the large majority (81.1%) of episodes CSF cultures were obtained on the same day as the urine sample. Also, antimicrobial activity was detected in the CSF in only 9.9% of the episodes. The vast majority of these patients had a normal CSF WBC count, while only ten had CSF pleocytosis. In only four of these could no alternative diagnosis be established; in none of these patients were microscopy and biochemical findings characteristic of bacterial meningitis. By assessing these aspects in detail we have limited the number of potentially false-negative CSF culture results, which adds to the robustness of our data. Notably, only one previous study, which included only 106 patients, has attempted to identify patients who had received antibiotics prior to lumbar puncture [19].

### Conclusions

Identification of co-existing meningitis in children presenting with UTI is critically important, as failure to detect CNS infection can result in partial treatment of meningitis with potentially severe long-term consequences [8], [9], [31]. Our findings suggest that the risk of co-existing meningitis in infants with UTI under the age of one month is not insignificant. In contrast, outside the neonatal period this risk is small, indicating that a selective, rather than universal, approach to lumbar puncture is warranted.
